# Monitoring coastal pollution associated with the largest oil refinery complex of Venezuela

**DOI:** 10.7717/peerj.2171

**Published:** 2016-06-23

**Authors:** Aldo Croquer, David Bone, Carolina Bastidas, Ruth Ramos, Elia García

**Affiliations:** 1 Departamento de Estudios Ambientales, Universidad Simón Bolívar, Caracas, Venezuela; 2 Departamento de Biologiía de Organismos, Universidad Simón Bolívar, Caracas, Venezuela; 3 Centro de Estudios Ecotoxicológicos en Sistemas Marinos (CETOXMAR), Caracas, Venezuela; 4 Massachusetts Institute of Technology, Cambridge, Massachusetts, United States

**Keywords:** Heavy metals, TPH, Macrobenthos, Polychaetes, Oil refinery, Coastal pollution, Polychaetes

## Abstract

This study evaluated pollution levels in water and sediments of Península de Paraguaná and related these levels with benthic macrofauna along a coastal area where the largest Venezuelan oil refineries have operated over the past 60 years. For this, the concentration of heavy metals, of hydrocarbon compounds and the community structure of the macrobenthos were examined at 20 sites distributed along 40 km of coastline for six consecutive years, which included windy and calm seasons. The spatial variability of organic and inorganic compounds showed considerably high coastal pollution along the study area, across both years and seasons. The southern sites, closest to the refineries, had consistently higher concentrations of heavy metals and organic compounds in water and sediments when compared to those in the north. The benthic community was dominated by polychaetes at all sites, seasons and years, and their abundance and distribution were significantly correlated with physical and chemical characteristics of the sediments. Sites close to the oil refineries were consistently dominated by families known to tolerate xenobiotics, such as Capitellidae and Spionidae. The results from this study highlight the importance of continuing long-term environmental monitoring programs to assess the impact of effluent discharge and spill events from the oil refineries that operate in the western coast of Paraguaná, Venezuela.

## Introduction

Marine ecosystems are frequently exposed to myriad disturbances associated with the rapid development of industrial and urban activities. These activities often result in anthropogenic impacts produced by chronic or acute sources of pollution. An overwhelming amount of evidence indicates that chronic inputs of chemical contaminants such as hydrocarbons and trace metals from focal points have vast impacts on marine ecosystems (Kilemade et al., 2004; Kim et al., 1999; Kraemer, Choudhury & Kampa, 2001; Venturini & Tommasi, 2004; Venturini et al., 2008). This is particularly true for coastal marine ecosystems close to oil refineries, which introduce into the environment 45,000 to 180,000 metric tons/year of toxic xenobiotics, such as polycyclic aromatic hydrocarbons (PAHs) and sulfur/ammonium compounds (Wake, 2005; CONCAWE 2004; GESAMP, 2007). During normal operation, oil refineries also introduce a wide range of heavy metals which may have synergistic toxic effects with other xenobiotics. These elements, alone or combined, have negative effects at all levels of biological organization (Venturini et al., 2008), and the magnitude of these effects depend on factors such as volume discharged, chemical characteristics, proximity to other sources of pollution and their interaction with local environmental variables (CONCAWE 1979, with hydrocarbon contamination that have “normal” TOC in concert with sediment acid volatile sulphide levels (AVS = sulphide accumulation from metabolic processing of organic material) off the charts (McLusky, Bryant & Campbell, 1986; Neuberger, Achituv & García, 2005; Wake, 2005).

Coastal ecosystems are negatively impacted by toxic wastes dumped into the oceans, and in particular are benthic organisms which cannot escape from chemical pollution due their limited potential for movement compared to organisms in the water column (Wake, 2005; Tollefsen et al., 2006). Organisms that live on the ocean bottom respond to xenobiotics by burrowing, by developing physiological strategies to cope with their sublethal effects, or dying (Wake, 2005). As soft bottom organisms are highly susceptible to toxic compounds, their patterns of abundance and distribution change in response to the presence of xenobiotics, physical features of the sediments, organic enrichment and/or a combination of these three (Tsutsumi, 1990; Riera & de-la-Ossa-Carrero, 2014). Therefore, departures from expected spatial and temporal patterns have been widely used as an indicator of chemical pollution in soft bottom communities (Venturini & Tommasi, 2004; Venturini et al., 2008; Arvanitidis et al., 2009). Changes in their trophic structure have also been used as a good proxy for detecting impacts of pollution as they are indicative of shifts in ecosystem function (Boesch, 1972; Gaston, 1987; Muniz & Pires, 1999; Snelgrove & Butman, 1994; Venturini & Tommasi, 2004). Compared to other taxa, polychaetes are one of the most conspicuous organisms in soft bottom communities, showing a wide variety of feeding strategies and a broad range of tolerance to different xenobiotics among different families (Fauchald & Jumars, 1979). In consequence, the presence/absence of particular families of polychaetes can also be a powerful indicator of habitat degradation and chemical pollution (Gaston, 1987; Gray et al., 1988; Warwick, 1988a; Warwick, 1988b; Dauvin, Gomez Gesteira & Salvande Fraga, 2003; Venturini & Tommasi, 2004; Giangrande et al., 2005).

Venezuela has one of the largest capabilities for oil processing, with an estimated daily capacity of 1,167,000 bpd (i.e., 166,714 metric tons/day). The estimated annual discharge of effluents into the marine environment is about 6,541 metric tons/year of oil waste (GESAMP, 2007). The largest oil refineries of Venezuela are located along the Caribbean coast, primarily the western part, and especially on the Península de Paraguaná, where the largest refinery complex (Complejo de Refinación de Paraguaná, CRP) is located. The CRP comprises three oil refineries: (1) Bajo Grande in Maracaibo (Zulia state), whereas (2) Amuay and (3) Cardón are both located along the western coast of Península de Paraguaná (Falcón state). Combined, these three oil refineries have the highest oil processing capacity in the world. Operations at CRP-Amuay and CRP-Cardón started in 1949 with metals and hydrocarbons being chronically introduced into the marine environment from localized sources for over 60 years. Despite the potential environmental risk that these CRPs may represent for the environmental and local human societies, no baseline studies were available for the area previous to this study.

Península de Paraguaná has one third of the population of Falcón state (ca. 350,000 people), with the largest concentration along the west coast of the peninsula along with major ports, transportation hubs and numerous fisheries. With a goal of studying the potential environmental risk of the CRPs and their prospective impacts on the marine biodiversity and the human health of this region, this study aimed: (1) to detect levels of pollution of organic and inorganic compounds in water and sediments along a putative pollution gradient during six years, (2) to assess the seasonal and spatial patterns of trace metals and other xenobiotics, and (3) to evaluate the environmental pollution as an explanatory variable in the distribution of polychaetes along this coastline.

## Materials and Methods

### Study area

**Figure 1:**
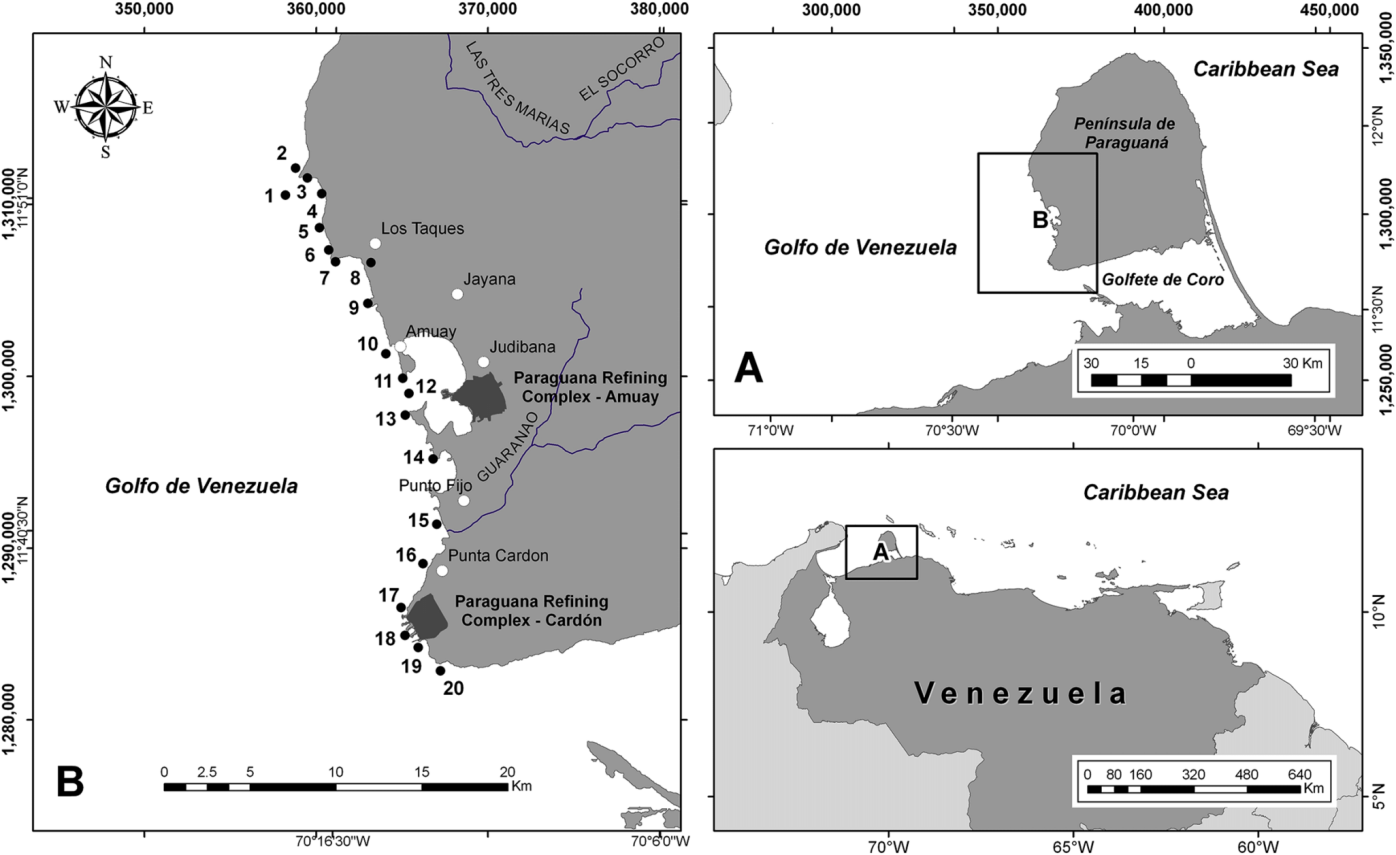
Study area. Spatial distribution of sampling sites across the western coast of Peninsula de Paraguaná, Venezuela. The numbers correspond to sampling sites.

Península de Paraguaná is located at the western coast of Venezuela and encompasses a total area of 3.405 km^2^ of arid terrestrial and coastal marine ecosystems (Fig. 1A). The western coast of this peninsula extends for about 100 km from Punta La Barra in the south up to Cabo San Roman in the north, comprising soft bottom and rocky shore communities (Carmona & Conde, 1989). The study area covered 40 km of coastline from Punta La Barra in the south to El Pico in the north. Within the study area, the northern sector is farther away from urban centers and potential sources of chemical pollution (sites 1–8, Fig. 1B), whereas the southern sector includes to oil refineries (i.e., CRP Amuay and CRP-Cardón, Fig. 1B), large ports and cities (sites 9–20, Fig. 1).

### Sampling design and sample collection

Sampling was conducted along 20 sites, from 2008 to 2014, and during two contrasting periods of wind activity and force each year: (1) January–June or windy season and (2) July–December or calmed season (Masciangioli & Febres, 2000). For each of the six years, sampling was replicated four times in each season (i.e., windy season: January, March, April and May; calmed season: July, September, October and December). Water and sediment samples were collected using a Niskin bottle and a Van Veen Grab (0.1 m^2^), respectively. Water samples were stored in coolers and transported to the laboratory to estimate the concentrations of trace metals, total suspended solids (TSS), total petroleum hydrocarbons (TPH) and total oil and grease (TOG) through standard analytical procedures described below. Sediment samples were also stored and transported to the laboratory to determine trace metals, TPH, total organic carbon (TOC), organic matter (OM), PAHs, and acid volatile sulfide (AVS).

### Chemical analyses of water and sediments

Water and sediment variables were determined by different EPA standardized procedures as briefly described here. The concentration of TSS was estimated following the EPA method number 340 (USEPA, 1993). From the TSS, the content of metals was determined according to the “Standard Methods for the Examination of Water and Wastewater” (ASTM, 2000), with an Inductive coupling plasma optical emission (ICP-OE) Optima 2100-DV. Trace metals in sediments were estimated from 1 g of sample following the same method, which allowed a recovery of 100, 96, 93, 87 and 82% for Cadmium (Cd), Zinc (Zn), Chrome (Cr), Nickel (Ni) and Lead (Pb), respectively.

The concentrations of TPH in water samples were determined according to EPA 1664 (USEPA, 2000). This method was also used for sediments but the extraction was improved by adding the same amount of acetone to the hexane solution. The concentrations of PAHs in sediments were determined according to EPA SW846 (USEPA, 2000), whilst the AVS was estimated with the SWEWW (2000) method. TOC and OM were determined in the sediments using the Walkley-Black (modified by Gross 1971) and Jackson (1970) methods, respectively. Granulometric analysis of sediments was also performed using standard procedures to determine the percentage of different particle fractions in the sediment (Folk & Ward, 1957).

### Characterization of the macrobenthic community

Samples to describe the benthic communities at each site were collected with a Van Veen Grab (0.1 m^2^) capable of penetrating at least 30 cm into the sediment. For this, three independent replicates spaced by 2–5 m were taken at each site and for every sampling period within each year. Every sample was labeled and kept in coolers before preservation. On land, samples were preserved in a 10% formalin solution and hand-washed through a 1 mm-mesh sieve to retain only the macrofauna. The remaining fraction was then observed with stereoscopes and microscopes when necessary. All organisms were identified and sorted into major taxonomic groups and their relative abundances was calculated as the total number of individuals within a taxonomical group divided by the total number of individuals (N). Amongst all taxonomic groups only polychaetes were classified into families, as they are known to be an exceptionally good indicator of environmental pollution (Dauvin, Gomez Gesteira & Salvande Fraga, 2003; Gray et al., 1988; Warwick, 1988a; Warwick, 1988b). While other taxa have also been recognized as pollution sentinels, polychaetes were preferred in this study for three reasons: (1) they were the dominant taxonomical group across all sites, years and seasons, further suggesting they could thrive with different levels of pollution, (2) this group was easily identifiable to family level with our expertise, and (3) the Family taxonomic level has been shown to be the best compromise for taxonomic resolution when an accurate identification is not achievable (Somerfield & Clarke, 1995; Bacci et al., 2009).

### Statistical analysis

Statistical analyses had two aims: (1) detecting spatial patterns of environmental pollutants associated to oil refineries in water and sediments between seasons, and (2) finding the best multivariate linear combination of environmental variables to predict polychaete abundance. For the first aim, Principal Component Analysis (PCA) was used from normalized data (i.e., each replicate was subtracted by the mean value and divided by the standard deviation) across years and seasons. Draftsman plots were used to eliminate highly correlated (ρ > 0.9) redundant variables from the analysis and to decide the proper transformations if needed. After variable selection, a PCA for water samples was performed with twelve of the original variables, whereas for sediments all the thirteen variables were used in the analysis. For water, log (x + 1) transformations were necessary for Cr, Pb and Hg, whereas for sediments, all metals were log (x + 1)-transformed due to their strong right-skewed distribution.

For the second aim, a canonical analysis of principal coordinates (CAP, Anderson, Gorley & Clarke, 2008) was done to obtain a constrained ordination of samples using PCA1 scores as a proxy for pollution concentration in the sediments (i.e., prediction variable for the abundance of polychaetes). A distance-based linear model (DstLM, Anderson, Gorley & Clarke, 2008) was then used to determine the contribution of each environmental variable alone (marginal test) and/or in linear combination (sequential test) to explain the variability of the abundance of polychaete families across sites and months. A distance-based redundancy analysis (DbRDA, Anderson, Gorley & Clarke, 2008) was also used to represent the best lineal model to explain the spatial distribution of polychaetes across sites for each year and season. All analyses were performed with PRIMER 6 + PERMANOVA.

## Results

### Chemistry of water and sediments

**Figure 2:**
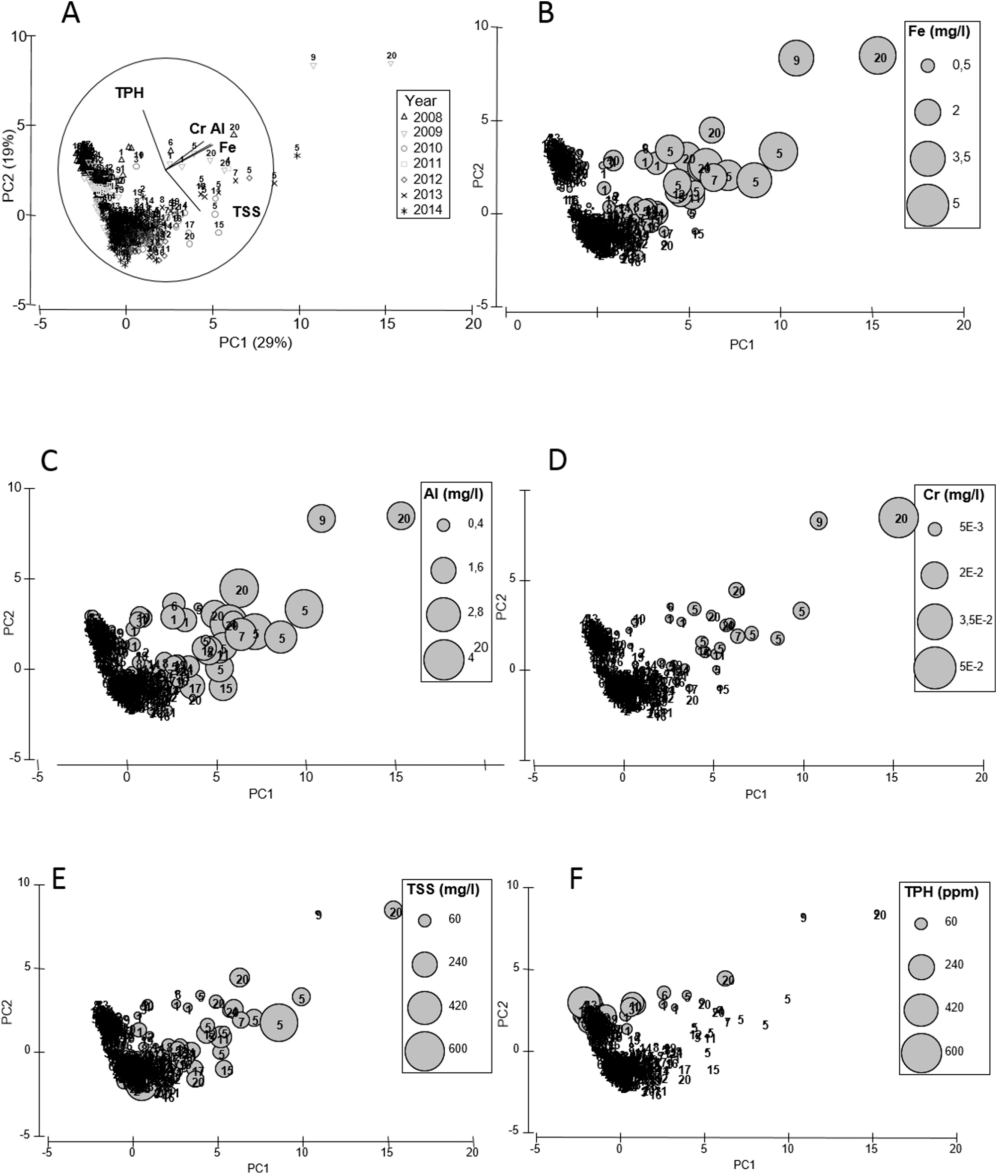
PCA water. Principal component analysis (PCA) for standardized environmental variables in the water column across sampling sites (labels) and years (legend) (A) and bubble plots for environmental variables relevant to each principal component (B–F).

**Figure 3:**
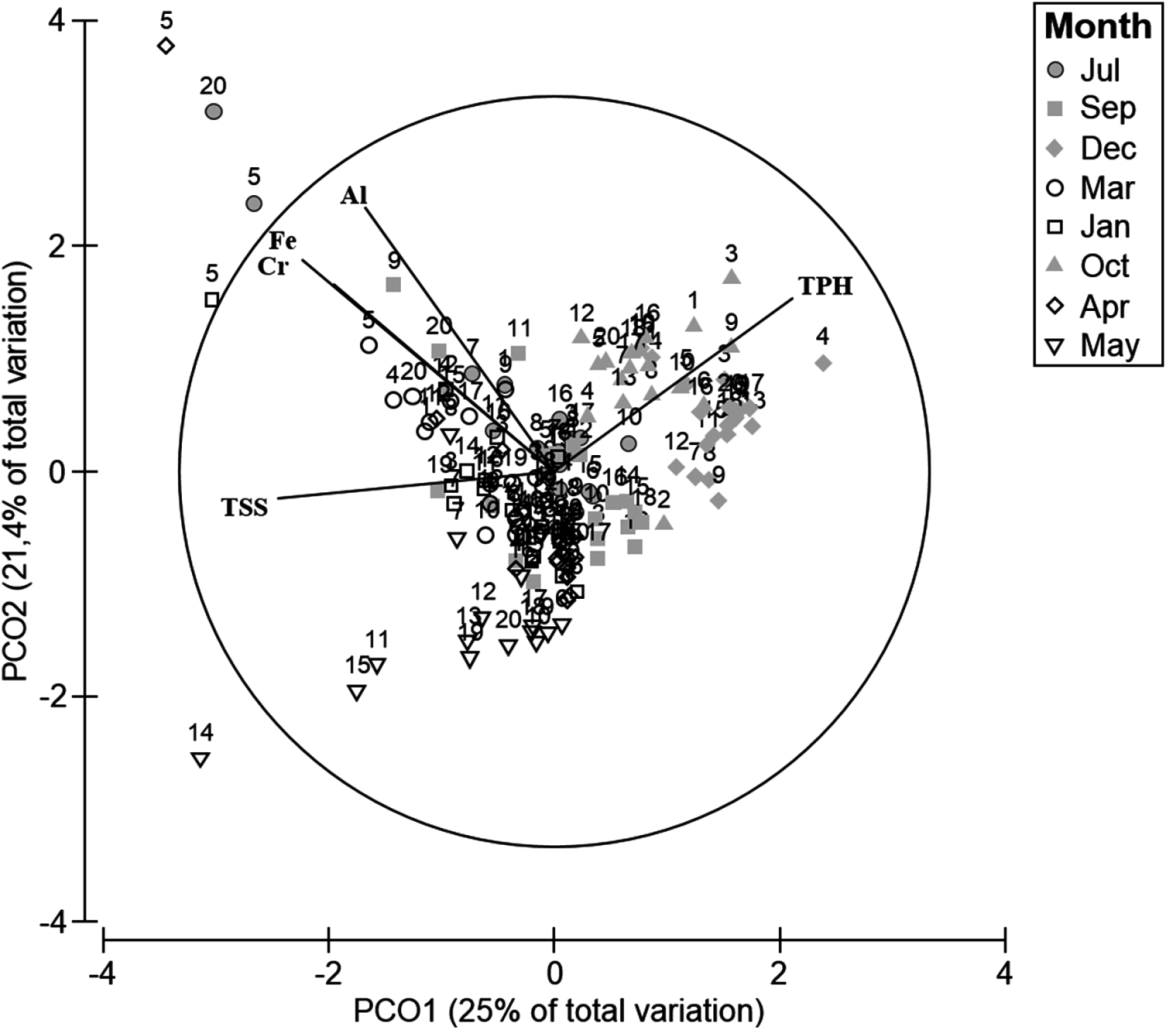
PCO water. Principal coordinate analysis (PCO) for centroids of standardized environmental variables in the water across sampling sites and seasons. Filled and empty symbols correspond to windy and calmed seasons, respectively.

Chemical analyses of the water revealed the presence of xenobiotics varying in time and space along the western coast of Paraguaná (Fig. 2). With 47% of the total variance explained by PCA1 and PCA2, samples closer to CRP oil refineries (i.e., sites 20 and 11) were ordinated along the PCA1 according to their higher concentrations of Fe, Al, Cr and TSS in water (Figs. 2A–2E). Samples from different years were ordinated along the PCA2 according to their concentrations of TPH (Fig. 2F). A clear seasonal pattern of the xenobiotic distribution across sampling sites was obtained with the PCO (Fig. 3). With 46% of the total variance explained between PCO1 and PCO2, calm season samples (i.e., July–December) showed the highest concentrations of TPH and heavy metals, whereas during the windy season samples had higher concentrations of TSS (Fig. 3). These results indicated that trace metals and residual hydrocarbons are present at detectable concentrations in water along the south-north gradient, and that this pattern is strongly seasonal.

**Figure 4:**
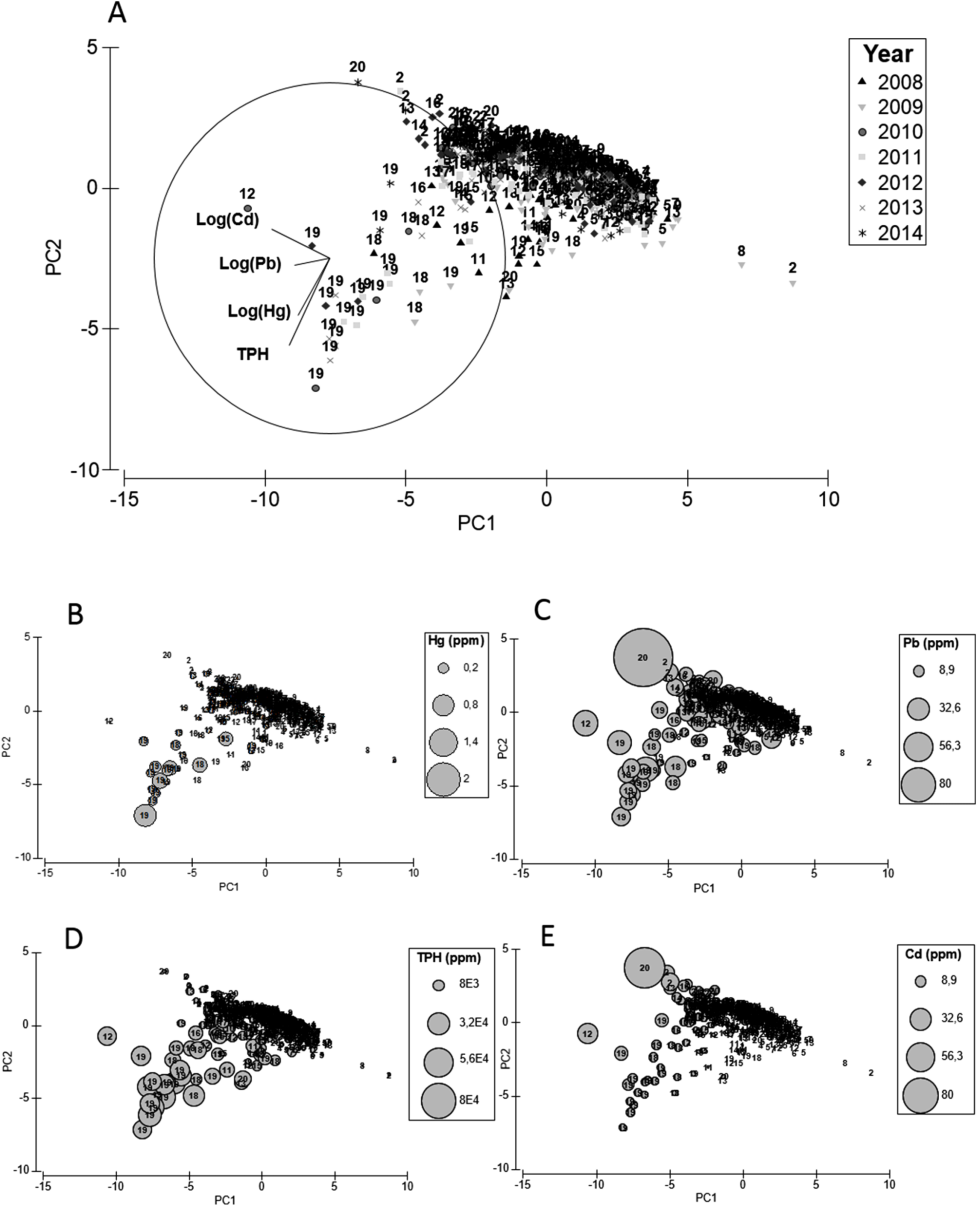
PCA sediments. Principal component analysis (PCA) for standardized environmental variables in the sediments across sampling sites (labels) and years (legend) (A) and bubble plots for environmental variables relevant to each principal component (B–E).

**Figure 5:**
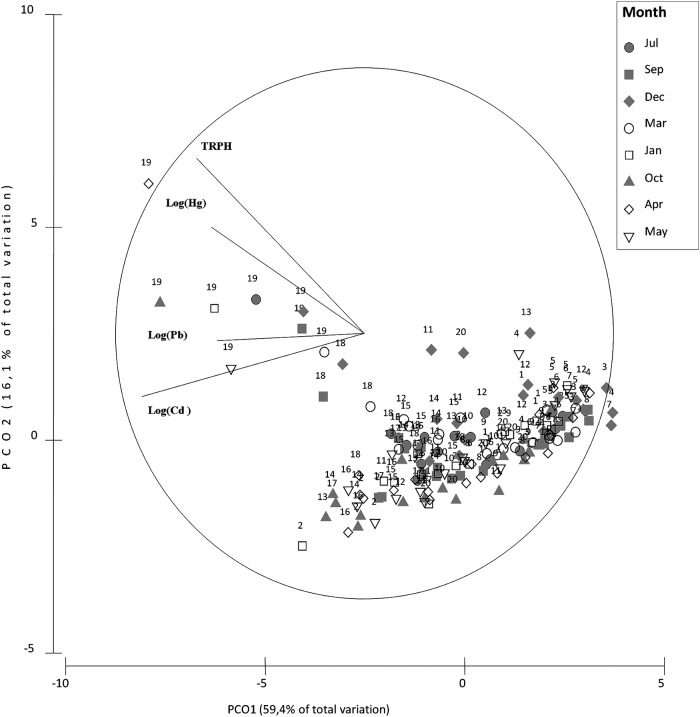
PCO sediments. Principal coordinate analysis (PCO) for centroids of standardized environmental variables in the sediments across sampling sites and seasons. Filled and empty symbols correspond to windy and calmed seasons, respectively.

Chemical analysis of the sediment showed higher concentrations of different types of pollutants in sites close to the oil refineries compared to sites located far away from them, this pattern being consistent across years (Table 1). For instance, in sites close to oil refineries, Hg and TPH were on average 4–12 fold higher than other sites. Similarly, the concentration of heavy metals such as Pb and Cd doubled at sites closer to the oil refineries compared with sites farther away (Table 1). The PCA supported the idea of a pollution gradient radiating out from refineries, with southern sites closer to CRP Cardón having the highest concentration of TPH and heavy metals across all years (Table 1; Fig. 4A). With 67% of the total explained by PCA1 and PCA2, sites 18 and 19 showed highest levels of Hg, TPH, Pb and Cd (Figs. 4B–4E). The PCO also showed a clear spatial gradient in terms of levels of pollution of these xenobiotics (Fig. 5). With a total of 75% of the total variance explained by PCO1 and PCO2, samples closer to the CRP Cardón (i.e., 19 and 18) consistently differed from all other sites regardless the season and year (Fig. 5). These results indicated that CRP Cardón has been the most important chronic source for the input of xenobiotics along the western coast of Paraguaná.

**Table 1:** Mean, standard deviation and ratios of concentrations of pollutants recorded in sites close to (sites 9–20) and far away from (1–8) the oil refineries.

Year	Pollutants	Sites close to oil refineries		Sites far away oil refineries		Ratio
		Avg/SD	Avg/SD			
2008	TPH	11.9	8.6	3.2	2.6	3.8
	Cd	3.2	1.1	1.7	1.6	2.0
	Hg	8.8	7.0	2.4	0.9	3.7
	Pb	3.5	1.4	1.4	1.3	2.5
2009	TPH	13.4	12.4	1.1	0.3	12.2
	Cd	3.5	1.6	1.8	1.1	1.9
	Hg	5.1	8.4	1.3	0.7	4.1
	Pb	6.0	3.6	2.7	0.9	2.2
2010	TPH	12.1	17.9	1.8	0.5	6.8
	Cd	4.7	4.9	1.9	1.9	2.5
	Hg	13.2	27.8	1.8	1.7	7.2
	Pb	6.8	4.7	2.4	1.2	2.9
2011	TPH	13.6	23.7	4.2	0.4	3.3
	Cd	4.0	2.3	2.8	3.5	1.4
	Hg	10.6	16.5	0.9	1.1	12.0
	Pb	4.6	3.7	3.4	3.0	1.4
2012	TPH	14.4	26.3	2.9	0.5	4.9
	Cd	7.1	3.8	3.0	3.0	2.4
	Hg	11.2	15.0	1.2	0.6	9.5
	Pb	6.5	6.0	2.1	2.1	3.1
2013	TPH	18.7	26.4	4.7	2.0	3.9
	Cd	4.9	2.8	2.5	2.5	2.0
	Hg	9.6	14.6	1.2	0.9	8.1
	Pb	5.0	4.9	2.9	2.9	1.7
2014	TPH	5.9	8.1	1.3	0.3	4.5
	Cd	12.8	28.9	6.2	7.2	2.1
	Hg	4.0	4.5	0.5	0.4	8.9
	Pb	12.0	29.0	4.9	4.3	2.4

### Relationships between environmental gradients and the abundance of polychaetes

The macrobenthic community along the coast of Paraguaná was consistently composed of thirteen major taxonomical groups during the sampling period (Table 2). From these groups, polychaetes accounted for 56–86% of the total abundance, whereas crustaceans represented 5–41% and mollusks 1–45%. Other groups such as Nermetea, Sipunculida and Echinodermata had less than 5% in terms of relative abundance (Table 2). Clearly, polychaetes were the most abundant and diverse group at all sites, seasons and years, with a total of 42 families observed; however, polychaete communities were dominated by only a few families (Table 3). The two families, Capitellidae and Spionidae, were the most conspicuous across sites, seasons and years, both accounting for 7–67% of the total relative abundance (Table 3). Other families such as Nereididae, Lumbrineridae and Sabellidae were also common across sites, seasons and years but represented less than 1–20% of the total abundance (Table 3). The large but related spatial variability of heavy metals, hydrocarbon compounds and polychaetes observed in this study, suggest that a combination of anthropogenic and environmental conditions prevailing at each site largely account for the distribution of polychaetes along the western coast of Paraguaná.

**Table 2:** Total relative abundance (%) of major taxonomic groups recorded across years and seasons along the western coast of Península de Paraguaná from 2008 to 2014.

Taxonomic groups	2008	2009		2010		2011		2012		2013		2014
	C	C	W	C	W	C	W	C	W	C	W	W
Polychaete	72.4	70.3	66.0	33.4	79.6	52.6	86.4	56.4	79.8	56.6	78.0	74.9
Mollusk	11.3	13.1	16.2	45.0	8.0	1.4	6.5	13.7	6.2	7.6	8.4	10.6
Crustacean	8.4	9.9	12.4	6.7	5.7	41.4	4.7	10.7	10.4	28.5	11.5	9.1
Sipunculida	4.7	2.7	2.4	6.0	1.0	0.7	1.0	6.9	1.0	1.6	0.5	3.5
Amphioxus	1.5	1.8	1.4	4.7	1.8	1.2	0.8	2.7	0.0	0.5	0.5	0.7
Others	1.7	2.3	1.6	4.2	3.9	2.7	0.5	9.6	2.5	5.2	1.2	1.1

**Table 3:** Total relative abundance (%) of major polychaete families recorded across years and seasons along the western coast of Península de Paraguaná from 2008 to 2014.

Polychaete families	2008	2009		2010		2011		2012		2013		2014
	C	C	W	C	W	C	W	C	W	C	W	W
Capitellidae	14.5	57.1	31.6	2.9	67.5	22.0	33.8	20.7	50.0	26.8	30.8	39.4
Saccocirridae	0.0	0.0	0.0	0.0	0.0	58.8	0.0	10.7	29.6	9.1	32.4	9.5
Spionidae	33.2	7.3	18.1	12.9	12.8	2.0	38.7	11.3	6.7	11.1	8.1	11.2
Nereididae	6.1	8.0	12.5	16.7	3.5	3.4	5.3	12.2	3.6	5.3	2.2	5.8
Lumbrineridae	8.5	6.3	8.9	20.0	2.3	3.4	6.0	4.2	1.4	8.2	3.0	5.2
Sabellidae	13.9	3.1	3.9	6.3	3.3	1.9	2.2	4.2	1.1	5.1	5.4	4.3
Syllidae	2.5	0.5	1.2	7.9	1.1	2.2	0.0	7.0	1.8	15.9	1.4	3.3
Opheliidae	5.3	3.0	3.6	4.2	2.0	1.1	4.2	3.4	0.5	0.0	3.5	0.0
Cirratulidae	3.5	2.8	1.7	0.8	1.5	0.4	1.1	8.2	0.9	2.1	2.8	1.5
Glyceridae	1.0	0.2	0.0	5.8	2.0	0.9	3.8	2.0	0.9	2.3	2.4	3.3
Onuphidae	2.6	0.5	7.0	0.0	0.2	0.1	2.0	1.1	0.2	1.0	0.6	1.4
Others	8.7	11.1	11.6	22.5	3.8	4.0	3.0	15.1	3.2	13.2	7.4	15.1

**Figure 6:**
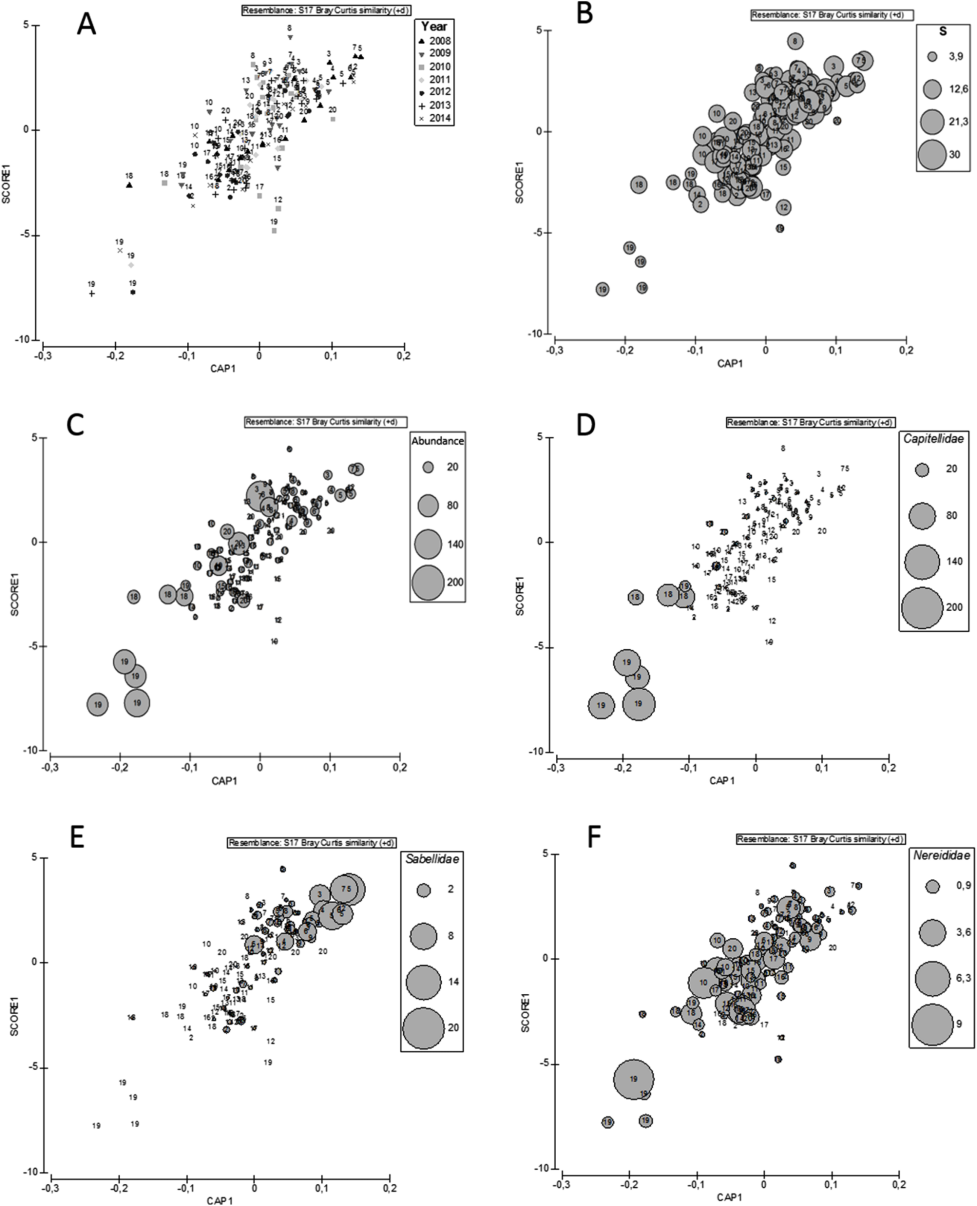
CAP. Canonical analysis of principal coordinates (CAP) showing the constrained ordination of samples using PCA1 scores from the PCA performed for the sediments as explanatory variable. Bubbles represent the relative abundance of polychaete families along the environmental gradient.

When examining the best linear combination of environmental variables as predictors of polychaete abundance, a strong and significant canonical correlation was found between PCA1 scores and the abundance of polychaetes across sites, seasons and years (r = 0.77, r^2^ = 0.61, p < 0.05; Fig. 6A). This result corroborates the idea that the abundance of polychaetes changes according to a gradient of environmental pollutants in sediment, radiating away from to the oil refineries. Compared to sites farther away, sites closer to the oil refineries (e.g., 18 and 19) showed a lower number of families (Fig. 6B) and higher abundances (Fig. 6C), further suggesting that few polychaete families might thrive in highly-polluted areas. In fact, some families had distribution patterns restricted to sites with highest levels of pollution (e.g. Capitellidae, Fig. 6D), while some families were most likely found in sites with lower levels of contamination (e.g. Sabellidae, Fig. 6E) and families such as Nereididae ha a broad range of spatial distribution regardless the level of pollution (Fig. 6F).

**Figure 7:**
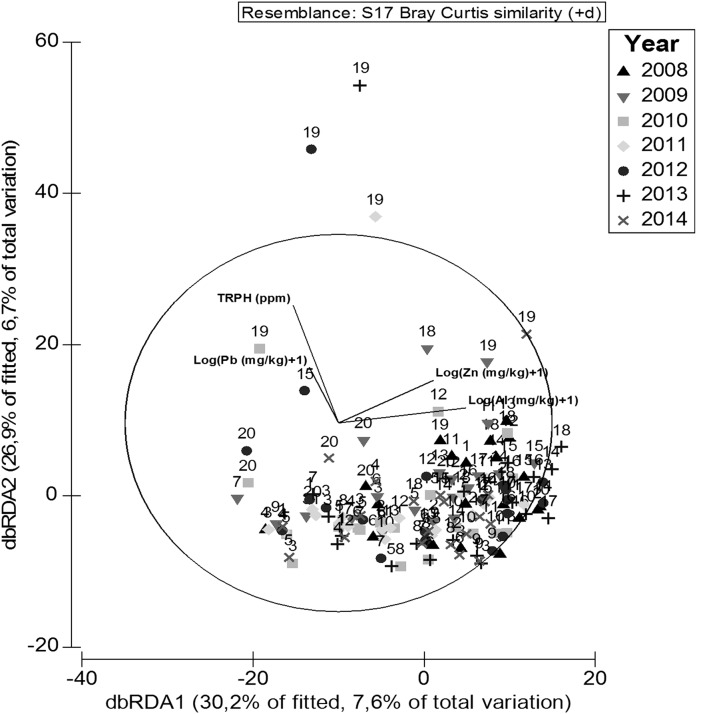
DstLM. Distance-based-Redundancy Analysis (DbRDA) showing the variables that better explained the ordination of samples based on results from the distance-based linear model (DstLM).

Our results indicated that nine (OM, TOC, OG, TPH, Cd, Cr, Fe, V and Al) out of thirteen explanatory environmental variables examined in the linear models were significantly correlated with the spatial pattern of polychaetes (Table 4). The combination of these environmental variables explained 25% (r^2^ = 0.60) of the total variability of polychaetes across years, seasons and sites. The distance-based redundancy analysis showed that 57% of the multivariate variation of the polychaetes was explained by the two first RDA axes (Fig. 7), further corroborating that the community of polychaetes was significantly influenced by the spatial and temporal distribution of the xenobiotics along the western coast of Paraguaná.

**Table 4:** Distance-based Linear Model (DstLM). Sum of Squares, Pseudo F, total variance explained (r^2^), and individual contribution of each environmental variable to explain the variability of the polychaete assemblages across sites and years.

Variable	SS (trace)	Pseudo-F	p-level	(r^2^)
M.O. (%)	2,448.8	2.1	0.038	0.015
C.O.T. (%)	6,156.1	5.3	0.001	0.037
Aceites (ppm)	8,995.3	7.9	0.001	0.054
TRPH (ppm)	9,813.6	8.7	0.001	0.059
Log(Cd (mg/kg) + 1)	7,186.0	6.3	0.001	0.043
Log(Cr (mg/kg) + 1)	8,952.0	7.9	0.001	0.054
Log(Pb (mg/kg) + 1)	5,595.1	4.8	0.002	0.034
Log(Ni (mg/kg) + 1)	8,923.9	7.9	0.001	0.054
Log(Zn (mg/kg) + 1)	10,149.0	9.0	0.001	0.061
Log(Fe (mg/kg) + 1)	8,124.1	7.1	0.001	0.049
Log(V (mg/kg) + 1)	6,839.7	5.9	0.001	0.041
Log(Hg (ppm) + 1)	7,029.4	6.1	0.001	0.042
Log(Al (mg/kg) + 1)	9,824.6	8.7	0.001	0.059
Total Variance explained (%)				60.04

## Discussion

### Spatial and temporal patterns of chemical compounds in water and sediments

Our results indicate that the high level of pollution in water and sediment along the western coast of Paraguaná is consistent with the long-term activities of oil refineries in the area. In water we found a strong seasonal correlation with higher levels of pollution during the calm (rainy) season in comparison to the dry season, for all years. During rainy months, terrestrial runoff increases and likely contributes to more pollutants reaching coastal waters close to the CRPs. Moreover, PDVSA (2014) reported hydrocarbon spills close to the oil refineries during the rainy seasons of 2008, 2010, and 2014.

During the windy (and drier) months, water had lower pollutant concentrations but higher levels of TSS. This result suggests that pollutants accumulated in the sediments might disperse during the windy season in the water column. Of particular concern are those sediments near the CRP oil refineries, which seemed to be a chronic source of pollution for the eastern coast of Paraguaná. While only scant oceanographic and atmospheric data are available locally to understand how these organic and inorganic compounds disperse in the water column, it is well known that coastal processes driven by winds, precipitation and terrestrial runoff influence dispersal patterns of trace metals and other xenobiotic compounds along continental shores (Dassenakis et al., 2006; Gavriil & Angelidis, 2005; Lee et al., 2008).

In waters of the west coast of Península de Paraguanà, four out of the six metals examined were in higher concentrations than in the east coast of this peninsula (Pb = 0.08, Ni = 0.07, Cd = 0.007 and Cr = 0.005 mg/l, García et al., 2008; García et al., 2011). Also, all heavy metal concentrations found in this study exceeded the NOAA’s levels considered capable of affecting fauna (Buchman, 2008). Mercury concentrations were also above the limits of chronic pollution for marine surface waters (i.e., 0, 94 μg/l, Buchman, 2008), particularly in sites close to oil refineries. We found similar levels of pollution between the west coast of Paraguaná and other sites under the influence of oil refineries in the Baltic Sea (Dassenakis et al., 2006). Nonetheless, the concentrations of heavy metals found in waters of Paraguanà were lower than those at Tinto and Odiel rivers in Huelva (Spain), one of the most metallic polluted estuaries in Europe (Vicente-Martorell et al., 2009).

In this study, concentrations of TPH found in waters were similar to others reported in areas under the influence of oil refineries (Chouksey, Kadam & Zingde, 2004; Al-Imarah & Ali, 2010; Li et al., 2010). On average, the concentrations of TPH along the west coast of Paraguaná exceeded by 77 fold the concentration established as safe for discharge in Venezuelan coastal waters (Ministerio del Ambiente y de los Recursos Naturales, Venezuela, 1995: 23 mg/l). Levels of pollution found in sites close to Cardón and Amuay oil refineries were also above standards limits for fisheries and aquatic life of the World Health Organization (WHO) (Chapman & Kimstach, 1996). Despite high levels of contaminants, TPH concentrations decreased in the water column from 2008 to 2014, as shown by the PCA analysis. This pattern might be explained either by the consecutive deployment of physical barriers aimed to control and retain oil spills during 3 consecutive years (2011–2014) and/or by the 8-month suspension of operations of the CRP-Amuay oil refineries in 2012 (PDVSA, 2014).

Trace metals and residual hydrocarbons were more abundant in muddy sediments, primarily in sites nearby the oil refineries, whereas sites farther away, with coarser sediments, had lower concentrations of pollutants. This result suggests that the spatial distribution of pollutants along the coast of Paraguaná is strongly influenced by the proximity to oil refineries but also by the features of the sediments. In other instances, pollution gradients set up through several variables such as temperature, light, nutrients, oxygen concentration, bio availability of metals and other biological processes (Bajt, 2012).

Most heavy metal concentration was below the minimum value of the Effect Range Low (ERL, i.e., the concentration below which the effects are rarely observed or predicted among sensitive life stages or species of biota) and the Effect Range Median levels of toxicity (ERM, i.e., the concentration above which effects are frequently or always observed among most species of biota). The results also indicated that ERL was above the levels allowed by NOAA only for Cd (Buchman, 2008). High enrichment factors for heavy metals (i.e., a normalized ratio of heavy metal content in a sample with respect to a reference concentration that is considered to act as a “proxy” for the clay content, Ravichandran et al., 1995) suggests that these metals are likely to be introduced in the environment by anthropogenic inputs rather than by natural sources alone (see Supplementary Table 5: enrichment factors for five heavy metals monitored across sites and years). Chemical and physical features of sediments vary depending on coastal geomorphology and the level of human impacts (Muniz, Venturini & Martínez, 2002; Tolosa et al., 2005; Venturini & Tommasi, 2004; Venturini et al., 2008). However, it is generally accepted that increasing levels of contamination often found nearby oil refineries and close to their effluents are largely due to human-related activities (Le Dréau et al., 1997; Wake, 2005), supported in this study by enrichment factors larger than one.

We found a clear gradient of pollution for TPH and heavy metal concentrations, with increasing values closer to the oil refineries. In particular, sites 18–20 which are located nearby to CRP-Cardón, showed levels of TPH pollution exceeding the national permitted standards (i.e., Ministerio del Ambiente y de los Recursos Naturales, Venezuela, 1995). Furthermore, the chronic toxicity of sediments polluted with TPHs and collected in the vicinities of CRP-Cardón was recently tested throughout bioassays using the polychaete *Scolelepis texana*. The results showed significantly higher levels of toxicity for sediments collected from sites 19, 18 and 20 compared to sediments collected far away (Ramos, Bastidas & García, 2012).

Close to the CRP, the concentration of Pb, Cd, Cr, Ni and Zn were similar to the ones reported in areas of high industrial, urban and coastal economic activities elsewhere (e.g. Acosta et al., 2002; Muniz, Venturini & Martínez, 2002; Kilemade et al., 2004; Idris, 2008). In other areas of the Venezuelan east coast, where other oil refineries are located, levels of Cr have been found in similar concentrations compared to CRP-Paraguaná (17.43 vs. 17.5 mg/kg), whereas Cd (1.2 vs. 2.7 mg/kg) and Pb were lower (14.77 vs. 29.8 mg/kg), and Zn was much higher (2,613.52 vs. 29.4 mg/kg, Martínez, Rodríguez & Senior, 2002). Higher concentrations of Zn at these other areas were attributed to the influence of anthropogenic sources such as the periodic discharge of contaminated rivers (Martínez, Rodríguez & Senior, 2002). In sediments collected in nearby areas with no oil refinery influence (Morrocoy National Park), concentrations of Cd, Cr, Ni and Pb were lower than those reported for Paraguaná (Caldera, Gutiérrez & Polanco, 2005; García et al., 2008; García et al., 2011). Similarly, in Plataforma Deltana, an eastern region with no direct influence of oil refineries, low concentrations of Cr, Cd and Ni (i.e., 33–45, 0.60 and 19.00–34.00 mg/kg, respectively), TPH (8.70–14.10 mg/kg) and PAHs (8.90–31.30 mg/kg) were also reported (Martín et al., 2007). In other countries, Venturini & Tommasi (2004) found up to 4.163 mg/kg of PAHs at Todos os Santos (Brazil), whilst PAHs in sediments along the Montevideo coast (Uruguay) reached up to 11.130 mg/kg (Muniz, Venturini & Martínez, 2002). In another study conducted at Port Cork, Irlanda, Kilemade et al. (2004) reported a narrow range of concentrations of PAH (0.528–2.878 mg/kg) compared to the ones reported in Paraguaná. Our study, therefore indicate that CRP-Cardón is a heavily-polluted area which exceeds by an order of magnitude (i.e., 2.500 ppm) the concentration of TPH reported in other oil refineries overseas (e.g. the Persian Gulf: 200 ppm, Tehrani et al., 2013). In general, our results indicated that levels of pollution of particular contaminants such as Hg, Pb, Cd and TPH in sites close to the oil refineries that have been operating in the area for decades are above local and international recommended standards. This suggests there is a severe problem of chronic pollution along the western coast of Paraguaná which increases nearby the oil refineries.

### Community structure of macrobenthos as environmental indicator of pollution

Polychaetes were the dominant group of the macrobenthos at all sites, years and seasons with a total of 42 families observed during the study period. Families Capitellidae and Spionidae accounted for 60% of the total abundance of the community. These two families, together with Nereidae were also the most frequent across sites, seasons and years as reported by Brett, Bone & Lopez-Ordaz (2013). A significant and strong canonical correlation was found between the abundance of polychaete families and the chemical and physical features of the sediments. The results indicated that in sites close to the oil refineries, Spionidae and Capitellidae consistently dominated polluted sediments as it has been reported for other areas (Gaston, 1987; Snelgrove & Butman, 1994). Other families such as Sabellidae, Syllidae, Glyceridae, and Opheliidae were more abundant in the northern sites, far away from the oil refineries. While the distribution, growth rates and the abundance of polychaetes such as Capitellidae have been shown to be affected by organic enrichment (Ansari, Igole & Parulekar, 1986; Tsutsumi, 1990; Tsutsumi et al., 1990, Riera & de-la-Ossa-Carrero, 2014), we found no statistical evidence to show that TOC and OM directly explained the distribution of these organisms across sites throughout the years. However, the results suggested that both; TOC and OM may have a primary role in determining the availability of hydrophobic pollutants as reported in other studies (e.g. Eggleton & Thomas, 2004). In fact, we found that most polluted sites along Paraguaná were also characterized by muddy, organically enriched sediments. Thus, the distribution of certain polychaete families along the western coast of Paraguaná may be a consequence of both levels of pollution and organic enrichment.

It is well established that TOC and OM are not always the best proxies of organic enrichment. Variables such as AVS may also provide useful information to interpret changes in the distribution of organisms across environmental gradients as rates of sulfate reduction increase in sediments enriched with TOC and OM (Como et al., 2007). While the AVS were determined in our study, this variable was not included in the multivariate analysis because there were too many missing data and probably as a result the correlation between AVS, TOC and OM was weak (r = 0.19 and 0.20, respectively). On the other hand, the concentration of AVS normally increases in muddy sediments with high levels of TPH (Shin, Pardue & Jackson, 2000), such as those observed close to the oil refineries in Paraguaná. We found a significant positive and weak correlation (r = 0.49, p < 0.05) between AVS and TPH when data for both variables were available. This result suggests that AVS might have a primary role in determining changes in the composition of the polychaete community across the environmental pollution gradient observed along the western coast of Paraguaná.

The proliferation of oil-consuming bacteria are also know to be a primary cause of organic enrichment and some of these bacteria may change the granulometry of the sediments, particularly in chronically-polluted areas as those close to the oil refineries of Paraguaná (Thiyagarajan et al., 2010). Unfortunately, our monitoring program did not take into account bacterial counts or their metabolic activity. In the future, the role of bacteria on organic enrichment and the features of the sediments should be addressed.

In general, we found a higher number of polychaete families in the northern area, where sandy sediments, with lower levels of pollutants, were found. These results indicated that the gradient of pollution observed along the coast of Paraguaná affects the spatial distribution of these organisms. Similar results have been reported by Roth & Wilson (1998) at Dublin Bay (Ireland) and by Venturini & Tommasi (2004) and Venturini et al. (2008) at Todos Os Santos Bay (Brazil). In these studies, the impact of oil contamination on benthic communities was assessed in areas under the influence of oil refineries or industries, showing that the macrofauna distribution is strongly determined by pollution gradients. In the particular case of Todos Os Santos Bay, chronic oil pollution has been correlated with a significant reduction in the number of macrobenthic species and especially of polychaetes. Furthermore, in the northern sector of this bay, a peak of abundance of few families such as Capitellidae and Spionidae was observed. There is strong evidence indicating that hydrocarbons, such as the ones recorded in our study, have detrimental effects on population size and reproductive potential of polychaetes; however, some species are able to degrade and excrete these compounds (Forbes, Forbes & Holmer, 1996). Thus, the dominance of polychaetes across years and seasons, particularly in highly polluted areas of the western coast of Paraguaná may be related to their capacity to cope with xenobiotic compounds. The Saccocirridae, a family associated with coarse sediments, was also abundant accounting for 9–58% of the total abundance across seasons from 2011 to 2014 (Table 3). This might be explained by changes in the sedimentary regimes observed in sites 1, 7 and 20. During these years, coarser grain sizes became more abundant in sediment samples collected from these three sites.

The Family taxonomic rank for identification of organisms has been supported as sufficient and adequate in monitoring the pollution effects on soft-bottom environments (Muniz & Pires-Vannin, 2005; Bacci et al., 2009; Musco et al., 2009). Particularly, polychaetes have been identified by several authors as a group of marine invertebrates with a fast response to environmental disturbances; thus, these organisms have been widely used as indicators of environmental pollution and habitat degradation (Giangrande et al., 2005). The success of Spionidae and Capitellidae to colonize highly polluted environments has been explained either by opportunistic attributes in their life histories, such as short-life cycles and high recruitment rates (Gray, 1989; Dean, 2008; Dahl Lücke & Johnson, 2009), or by their capacities to tolerate the toxic effects of targeted xenobiotics. This tolerance may occur through different mechanisms such as the production of defense enzymes such as cytochrome P450-P420, cytochrome b5, and glutathione-Sulfur transferase, among others (Driscoll & McElroy, 1996; Lee, 1998; Jørgensen et al., 2008). These mechanisms were recently described for Spionidae polychaetes collected close to the area of influence of CRP-Cardón (Manuitt, 2011).

To conclude, we found evidence of pollution along the western coast of Paraguaná. A spatial gradient was found for pollutants, as sites in the southern sector, closer to oil refineries, showed higher concentrations of heavy metals and organic compounds, both in water and sediments compared to northern sites. This spatial pattern was consistent for the six years examined. The fewer number of polychaete families and the dominance of Spionidae and Capitellidae in sites closer to the oil refineries, were highly correlated with sediment pollution, suggesting a higher tolerance of these families to the presence of xenobiotics compared to the other 40 families found. These results highlight the need for establishing long-term monitoring programs aimed at better understanding these ecosystems and help to control the discharges of effluents and spill events.

## Supplemental Information

10.7717/peerj.2171/supp-1 Supplemental Information 1 Supplementary table. Enrichment factors (EF) of several heavy metals recorded in ten sediments along the western cpst of Paraguaná. EFs were calculated using Fe as metal of reference.

10.7717/peerj.2171/supp-2 Supplemental Information 2 Raw data. Chemical variables measured in water and sediments across years, months and sites along the western coast of Paraguaná, Venezuela.

10.7717/peerj.2171/supp-3 Supplemental Information 3 Raw data. Major taxanomical groups recorded across years, sites and months along the western coast of Paraguná, Venezuela.

10.7717/peerj.2171/supp-4 Supplemental Information 4 Raw data. Polychaete families recorded across years, sites and months along the western coast of Paraguaná, Venezuela.

## Additional Information and Declarations

### Data Deposition

The following information was supplied regarding data availability: Raw data provided as Supplemental Dataset Files.
